# Supplementary material for: Effects of artificially-simulated acidification on potential soil nitrification activity and ammonia oxidizing microbial communities in greenhouse conditions
Source: PeerJ. 2022 Oct 3;10:e14088. doi: 10.7717/peerj.14088 (PMC9536323; doi:10.7717/peerj.14088)
Supplement: Supplemental Information 1 — In the table, an asterisk (*) indicates the p-value of correlation, and the number indicates the R-values of correlation; * 0.01 < p ≤ 0.05, ** 0.001 < p ≤ 0.01.SOM (Soil organic matter), TN (Total nitrogen), MBC (Soil microbial carbon), MBN (Soil microbial nitrogen). [file peerj-10-14088-s001.docx]

| Correlation coefficient | pH | SOM | TN | NH_4_^+^-N | NO_3_^-^-N | NH_3_ |
| --- | --- | --- | --- | --- | --- | --- |
| pH | - | 0.907** | 0.931** | -0.927** | 0.529* | 0.807** |
| SOM | 0.907** | - | 0.946** | -0.861** | 0.304 | 0.786** |
| TN | 0.931** | 0.946** | - | -0.815** | 0.312 | 0.864** |
| NH_4_^+^-N | -0.927** | -0.861** | -0.815** | - | -0.427 | -0.628* |
| NO_3_^-^-N | 0.529* | 0.304 | 0.312 | -0.427 | - | 0.198 |
| NH_3_ | 0.807** | 0.786** | 0.864** | -0.628* | 0.198 | - |
| Urease | 0.951** | 0.954** | 0.932** | -0.901** | 0.528* | 0.725** |
| Protease | 0.949** | 0.929** | 0.897** | -0.967** | 0.447 | 0.667** |
| MBN | 0.670** | 0.487 | 0.507 | -0.648** | 0.750** | 0.269 |
| MBC | 0.339 | 0.127 | 0.083 | -0.332 | 0.701** | -0.055 |
