# Supplementary material for: Effects of artificially-simulated acidification on potential soil nitrification activity and ammonia oxidizing microbial communities in greenhouse conditions
Source: PeerJ. 2022 Oct 3;10:e14088. doi: 10.7717/peerj.14088 (PMC9536323; doi:10.7717/peerj.14088)
Supplement: Supplemental Information 2 — In the table, an asterisk (*) indicates the p-value of correlation, and the number indicates the R-values of correlation; * 0.01 < p ≤ 0.05, ** 0.001 < p ≤ 0.01.SOM (Soil organic matter), TN (Total nitrogen), MBC (Soil microbial carbon), MBN (Soil microbial nitrogen), PNA (Potential nitrification activity). [file peerj-10-14088-s002.docx]

| Correlation coefficient | PNA |  | Abundance | |  | Diversity | |  | Tomato Plant | |
| --- | --- | --- | --- | --- | --- | --- | --- | --- | --- | --- |
|  |  |  | AOA | AOB |  | AOA | AOB |  | Total Biomass | Yield |
| pH | 0.888** |  | 0.960** | 0.605* |  | -0.386 | 0.919** |  | 0.151 | 0.591* |
| SOM | 0.859** |  | 0.950** | 0.634* |  | -0.456 | 0.723** |  | -0.007 | 0.505 |
| TN | 0.890** |  | 0.934** | 0.599* |  | -0.503 | 0.797** |  | -0.034 | 0.439 |
| NH_4_^+^-N | -0.924** |  | -0.949** | -.674** |  | 0.463 | -0.778** |  | -0.08 | -0.537* |
| NO_3_^-^-N | 0.273 |  | 0.346 | 0.474 |  | 0.35 | 0.722** |  | 0.813** | 0.786** |
| NH_3_ | 0.663** |  | 0.794** | 0.196 |  | -0.382 | 0.701** |  | -0.208 | 0.192 |
| MBN | 0.544* |  | 0.550* | 0.550* |  | -0.026 | 0.783** |  | 0.574* | 0.682** |
| MBC | 0.098 |  | 0.229 | 0.307 |  | 0.333 | 0.547* |  | 0.801** | 0.762** |
| Urease | 0.881** |  | 0.946** | 0.766** |  | -0.364 | 0.836** |  | 0.216 | 0.662** |
| Protease | 0.932** |  | 0.972** | 0.773** |  | -0.447 | 0.809** |  | 0.142 | 0.619* |
