# Supplementary material for: Effects of artificially-simulated acidification on potential soil nitrification activity and ammonia oxidizing microbial communities in greenhouse conditions
Source: PeerJ. 2022 Oct 3;10:e14088. doi: 10.7717/peerj.14088 (PMC9536323; doi:10.7717/peerj.14088)
Supplement: Supplemental Information 3 — Diversity was represented by the Shannon index and community composition was replaced by the PCA1 axis. In the table, an asterisk (*) indicates the p-value of correlation, and the number indicates the R-values of correlation; * 0.01 < p ≤ 0.05, ** 0.001 < p ≤ 0.01. PNA (Potential nitrification activity). [file peerj-10-14088-s003.docx]

|  |  | AOA |  |  |  | AOB |  |
| --- | --- | --- | --- | --- | --- | --- | --- |
|  | Abundance | Diversity | Composition |  | Abundance | Diversity | Composition |
| PNA | 0.926** | -0.630* | 0.791** |  | 0.698** | 0.703** | 0.438 |
