# Supplementary material for: Effects of artificially-simulated acidification on potential soil nitrification activity and ammonia oxidizing microbial communities in greenhouse conditions
Source: PeerJ. 2022 Oct 3;10:e14088. doi: 10.7717/peerj.14088 (PMC9536323; doi:10.7717/peerj.14088)
Supplement: Supplemental Information 4 [file peerj-10-14088-s004.docx]

|  | AOA | | | |  | AOB | | | |
| --- | --- | --- | --- | --- | --- | --- | --- | --- | --- |
|  | CAP1 | CAP2 | r^2^ | p-values |  | CAP1 | CAP2 | r^2^ | p-values |
| pH | -0.99421 | 0.107418 | 0.722868 | 0.002 |  | -0.83162 | -0.55535 | 0.827044 | 0.001 |
| TN | -0.9955 | 0.094814 | 0.824035 | 0.001 |  | -0.8589 | -0.51214 | 0.895073 | 0.001 |
| SOM | -0.99855 | 0.053903 | 0.622392 | 0.005 |  | -0.85291 | -0.52206 | 0.738976 | 0.001 |
| NH_4_^+^-N | 0.935036 | -0.35455 | 0.586237 | 0.004 |  | 0.676141 | 0.736773 | 0.736126 | 0.003 |
| NO_3_^-^-N | -0.71299 | 0.701171 | 0.057417 | 0.701 |  | -0.21291 | -0.97707 | 0.145804 | 0.398 |
| NH_3_ | -0.94496 | -0.32717 | 0.942568 | 0.001 |  | -0.99747 | -0.07111 | 0.943831 | 0.001 |
